# Anthracyclines induce early changes in left ventricular systolic and diastolic function: A single centre study

**DOI:** 10.1371/journal.pone.0175544

**Published:** 2017-04-13

**Authors:** Anita Boyd, Paul Stoodley, David Richards, Rina Hui, Paul Harnett, Kim Vo, Tom Marwick, Liza Thomas

**Affiliations:** 1 Westmead Private Cardiology, Westmead, NSW, Australia; 2 School of Medicine, Western Sydney University, Campbelltown, NSW, Australia; 3 Sydney South West Clinical School, University of New South Wales, Sydney, NSW, Australia; 4 Crown Princess Mary Cancer Centre, Westmead Hospital, Westmead, NSW, Australia; 5 Westmead Hospital and Westmead Clinical School, University of Sydney, Sydney, NSW, Australia; 6 Baker IDI, Melbourne, Victoria, Australia; 7 University of Tasmania, Tasmania, Australia; Scuola Superiore Sant'Anna, ITALY

## Abstract

**Aims:**

2 dimensional (2D) strain analysis detects subclinical left ventricular (LV) systolic dysfunction. Our aim was to evaluate changes in LV systolic and diastolic function in breast cancer patients early after anthracycline chemotherapy, and to identify predisposing factors.

**Methods and results:**

140 patients were assessed by detailed echocardiography before and within seven days post treatment. LV ejection fraction (LVEF), global longitudinal strain (GLS), strain rate and radial and circumferential strain were assessed. Additionally, left atrial volumes and LV diastolic parameters were evaluated. LVEF although reduced after treatment, remained within the normal range (60±3% vs. 59±3%, p = 0.04). Triplane GLS was significantly reduced after treatment (-20.0±1.6% vs. -19.1±1.8%, p<0.001). Subclinical LV dysfunction (>11% reduction in GLS compared to before therapy) occurred in 22% (29/135). Impaired diastolic function grade significantly increased from 46% to 57% (p<0.001) after treatment. Furthermore, diastolic dysfunction was more common in the subgroup group with reduced systolic GLS compared to those without changes in GLS (30% vs. 11%; p = 0.04). No risk factors or clinical parameters were associated with the development of subclinical LV dysfunction; however the percentage change in early diastolic strain rate and the E velocity were independent predictors of >11% reduction in GLS.

**Conclusion:**

Twenty two percent of patients had subclinical LV dysfunction by GLS, whilst none had cardiotoxicity defined by LVEF, demonstrating that GLS is more sensitive for detection of subclinical LV systolic dysfunction immediately after anthracycline therapy. Diastolic dysfunction increased, particularly in the group with reduced GLS, demonstrating the close pathophysiological relationship between systolic and diastolic function.

## Introduction

Anthracyclines are the cornerstone in breast cancer chemotherapy [1] with doxorubicin and epirubicin most frequently used [2]. They possess potent antitumour properties, a major factor in improved breast cancer survival [3]. However, anthracyclines are potentially cardiotoxic with cardiac dysfunction characterised by permanent and dose dependent myocardial damage [4]. The incidence of congestive heart failure or cardiac death in breast cancer patients receiving chemotherapy was 3% by 3 years compared to 1% in an age matched cohort not receiving chemotherapy [5]. The overall incidence of cardiotoxicity, defined as a reduction in left ventricular ejection fraction (LVEF), has been reported in up to 9% of patients receiving anthracyclines [6]. Furthermore, almost one third of patients receiving anthracyclines, trastuzumab or both, developed abnormal global longitudinal strain (GLS) at 6 months [7]. For this reason, breast cancer patients treated with anthracyclines require close evaluation of cardiac function after chemotherapy to enable early identification and treatment of cardiotoxicity [8, 9].

Echocardiography is the foundation for monitoring cardiotoxicity [10]. Historically, LVEF has been the key parameter for detecting cardiotoxicity; however, small changes in LVEF have low sensitivity given the variability in LVEF measurements [10]. More recently, 2D myocardial strain imaging has been shown to be more sensitive than LVEF in detecting LV systolic dysfunction following anthracyclines [10,11, 12].

While early changes in systolic function have been described, only limited studies with small sample sizes have assessed the effect of anthracycline chemotherapy on LV diastolic function, and particularly the relationship between systolic and diastolic function in this setting. Diastolic dysfunction may precede systolic dysfunction providing an early marker of cardiotoxicity and development of heart failure with relatively preserved systolic function.

We evaluated LV systolic and diastolic function in a large single centre cohort of chemotherapy naïve breast cancer patients before anthracycline treatment and within seven days after treatment, to evaluate myocardial dysfunction in a larger cohort than previously published reports. The main aim of this study was to evaluate and characterise the cardiotoxic effects of anthracyclines by the use of strain analysis and traditional echocardiographic measures. We also sought to examine segmental LV function and to identify clinical and echocardiographic predictors for the development of subclinical LV dysfunction.

## Methods

Study approval was obtained from the Committees for Human Research at Sydney West Area Health Service and the University of Sydney; all subjects provided written informed consent. 151 consecutive patients with histologically confirmed breast cancer were prospectively recruited. Anthracycline chemotherapy, either doxorubicin or epirubicin, was administered (4 to 6 cycles) as determined by the treating oncologist. No patients were receiving trastuzumab therapy at the time of this study. 35 patients went on to be treated with trastuzumab after the conclusion of this study. All patients underwent detailed clinical evaluation including cardiovascular history and risk factors (hypertension, diabetes, cholesterol, smoking history), cardioactive medications, family history, height, weight and blood pressure. Blood tests (haemoglobin, eGFR, creatinine) were performed before and after completion of anthracycline chemotherapy. Exclusion criteria included previous chemotherapy or radiation treatment, coronary artery disease, more than mild valvular disease, history of atrial or ventricular arrhythmias, an LVEF <50%, previous cardiac surgery or implanted devices.

All participants underwent a comprehensive transthoracic echocardiogram, according to established laboratory practice (Vivid 7 General Electric medical systems, Norway). Optimised imaging of the left ventricle (LV) was performed and all measurements were averaged over three consecutive cardiac cycles and offline analysis was performed using Echopac PC 6.1.0. (General Electric Vingmed, Norway). Baseline echocardiograms were performed at least one week prior to the commencement of anthracycline chemotherapy. Follow-up echocardiograms were performed within 7 days of completion of anthracycline chemotherapy (12 to 18 weeks following the baseline study) and prior to commencement of trastuzumab or thoracic radiation. Eleven patients were excluded due to inadequate image quality following left breast surgery; the remaining 140 patients were included in the analysis. All echocardiograms were performed with the patient in the left lateral position and images were obtained from the parasternal, apical and subcostal views.

### Left ventricular (LV) systolic function

LV volumes were measured from the apical 4 and 2-chamber views using the modified Simpson’s biplane method of discs, and LVEF was calculated [13]. Cardiac output was obtained by multiplying the Doppler calculated stroke volume by heart rate. Anthracycline related cardiac dysfunction was defined as an asymptomatic reduction in LVEF of >10% to a value of <53% as per guidelines [10]. LV mass was measured using the 2D linear cube method from the parasternal long axis view [13]. Pulsed wave Doppler tissue imaging was used to measure the average peak velocity in systole (S’) with the sample volume placed at the septal and lateral annulus.

**Fig 1:**
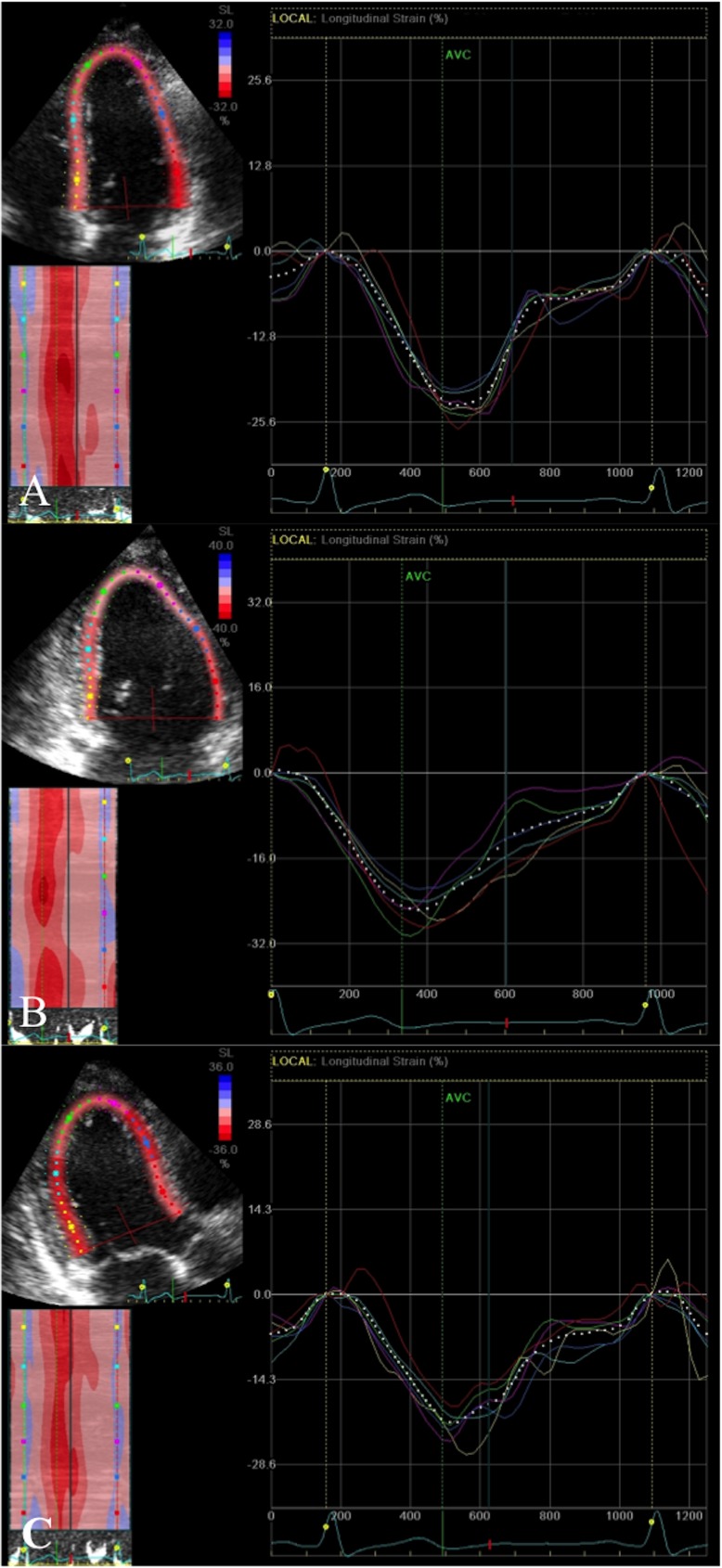
Global longitudinal strain. Peak systolic negative deflection from six segments (basal, mid and apical) from each view A. Four chamber view–septal and lateral walls; B. Two chamber view–inferior and anterior walls and C. Long axis view–posterior and anteroseptal walls. X-axis = Time (s); Y-axis = Strain (%).

**Fig 2:**
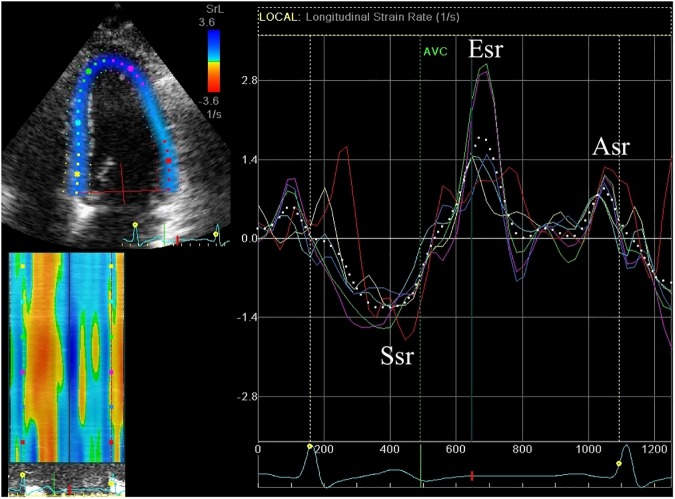
Strain rate. Systolic strain rate (Ssr), early diastolic strain rate (Esr) and late diastolic strain rate (Esr). X-axis = Time (s); Y-axis = Strain rate (s^-1^).

**Fig 3:**
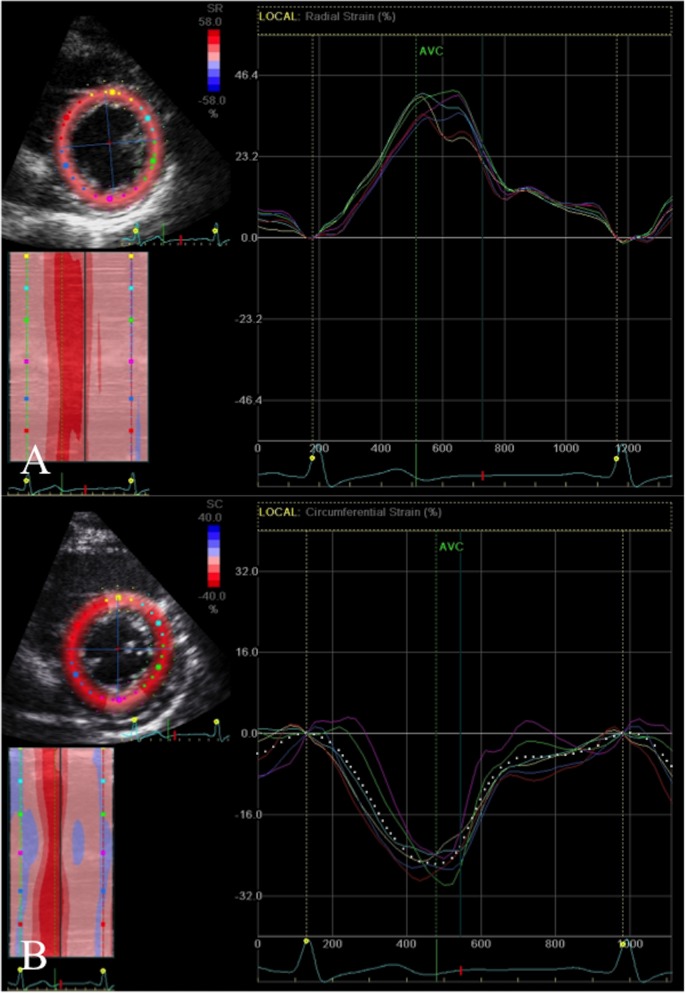
Radial and circumferential strain. A. Radial strain (peak systolic positive deflection) and B. Circumferential strain (peak systolic negative deflection) from the parasternal short axis view at the level of the papillary muscles (septal, anteroseptal, anterior, lateral, posterior, inferior segments). X-axis = Time (s); Y-axis = Strain (%).

2D strain analysis was used to measure global and regional longitudinal systolic strain from the apical four, two and three chamber views (basal, mid and apical segments from the septal, lateral, inferior, anterior, posterior and anteroseptal walls; (Fig 1), obtained at high frame rates (60-80fps) and optimised for 2D quality. The LV regions of interest were manually selected by marking the endocardial border. Measurements were only accepted if at least 4/6 segments from each apical view were adequately tracked. Systolic strain was measured as the peak negative strain during systole. Global biplane (12 segments from 4- and 2- chamber views) and triplane (18 segments from 4-, 2-, 3- chamber views longitudinal strain (GLS) was calculated as the average of the regional values. Subclinical cardiac dysfunction was defined as a reduction in GLS of >11% as previously reported [14]. Global systolic strain rate was calculated from the triplane apical segments (Fig 2). Global radial and circumferential strain were measured as the average of the six regional segments from the parasternal short axis view at the level of the papillary muscles (Fig 3).

### Left atrial (LA) volumes and diastolic function

LA maximum volume, LA minimal volume and LA pre ‘a’ wave volume (onset of P wave on ECG) were measured using the biplane area-length method. Phasic LA volumes including passive, conduit and active volumes and fractions were also calculated [15]. LA reservoir function occurs during systole and involves the storage of blood within the atria and is measured as the total emptying volume (LA maximum volume- LA minimal volume/ LA maximum volume). LA conduit function occurs during early diastole and involves the passive transfer of blood and is measured as the passive emptying volume (LA maximum volume- LA pre ‘a’ volume). LA active atrial function occurs during late diastole to augment ventricular filling and is measured as active emptying volume (LA pre ‘a’ volume–LA minimal volume). The LA conduit volume is measured as LV stroke volume–LA stroke volume (where LA stroke volume is LA maximum volume- LA minimal volume).

Transmitral inflow was obtained using pulsed wave Doppler. The peak velocity in early diastole (E wave velocity) and the peak velocity in late diastole (A wave velocity) and the E/A ratio were measured. The downward slope of the early diastolic E wave velocity was measured to obtain the deceleration time [15]. The active atrial emptying fraction was measured as the ratio of the A wave velocity time integral (VTI) to the total mitral inflow VTI [15]. Pulmonary vein peak systolic, diastolic and atrial reversal velocities were measured and peak systolic to peak diastolic velocity ratio obtained. Pulsed wave Doppler tissue imaging was used to measure the averaged peak velocity in early diastole (E’) and late diastole (A’) from the septal and lateral annulus. Global early diastolic strain rate (Esr) and late diastolic strain rate (Asr) were calculated from the 18 triplane segments (Fig 2). The E/E’ and E/Esr ratio were calculated as measures of LV end diastolic pressure.

LV diastolic dysfunction was stratified into four grades according to the ASE guidelines based on the E’ velocity, E/E’ ratio, E/A ratio, deceleration time and pulmonary vein flow as normal, impaired relaxation, pseudo normal filling or restrictive [15]. Normal diastolic function comprised of an E/A ratio > 1 with deceleration time <240 ms with normal E’ velocity and normal LA volume. The mildest form of diastolic dysfunction is impaired relaxation and was present if E/A ratio was < 1 and deceleration time exceed 240 ms. Pseudo normal diastolic dysfunction was present if E/ A ratio was > 1 and deceleration time was < 240 ms, but with low E’ velocity and an enlarged LA volume (> 34 mL/m^2^). The severest form of diastolic dysfunction is restrictive filling and was present if E/A ratio was >2 and / or deceleration time was < 140 ms [15].

### Statistical analysis

All continuous variables are expressed as a mean ± SD and categorical variables as a percentage. Paired t-tests were used to compare parameters before and after anthracycline therapy. Subgroup analysis was performed using unpaired t-tests. Non-parametric variables were examined by Chi square analysis. Pearson correlations were used to examine associations between continuous parameters. Multivariate analysis was performed by logistic regression backward stepwise likelihood ratio. Inter- and intra-observer variability of LVEF, triplane GLS and systolic strain rate were assessed on fifteen randomly selected patients by intraclass correlation coefficient. Data were analysed using SPSS version 21 (SPSS Inc, Chicago, Illinois), and considered significant if p < 0.05.

## Results

A total of 140 prospectively recruited patients were included in the final analysis; 99/140 (76%) received doxorubicin (419 ± 67 mg/m^2^) and 31/140 (24%) epirubicin (therapeutic equivalent dose 450± 136 mg/m^2^). Cardiovascular risk factors and cardioactive medications are listed in Table 1. Thirty six (26%) patients had more than one cardiac risk factor. No participant reported symptoms of cardiac failure during the chemotherapy.

**Table 1:** Patient cardiovascular risk factors.

Age (years)		52 ± 9 (range 33–77)
**Cardiovascular Risk factors**	Family history	45 (32%)
	Hypercholesterolaemia	26 (19%)
	Hypertension	31 (22%)
	Diabetes	8 (6%)
	Smoking	36 (26%)
**Cardioactive medications**	Beta blockers	2 (1%)
	Angiotensin converting enzyme inhibitors	7 (5%)
	Statins	13 (9%)
	Angiotensin II receptor blocker	18 (13%)
	Calcium channel blocker	10 (7%)
	Diuretics	3 (2%)

Haemoglobin was significantly reduced after anthracycline therapy (p<0.001), whilst estimated glomerular filtration rate (p = 0.53) and creatinine (p = 0.24) remained unchanged (Table 2).

**Table 2:** Patient clinical and left ventricular systolic echocardiographic parameters.

	Before anthracycline chemotherapy	After anthracycline chemotherapy
Haemoglobin (g/L)	131 ± 12	117 ± 12 *
eGFR (mL/min/1.73m^2^)	84 ± 9	85 ± 11
Creatinine (umol/L)	62 ± 10	61 ± 15
BSA (m^2^)	1.8 ± 0.2	1.8 ± 0.2
Heart rate (bpm)	71 ± 11	78 ± 11 *
MAP (mmHg)	90 ± 10	90 ±10
LV mass (g/m^2^)	75 ± 17	78 ± 16
Cardiac output (L/min)	5.0 ±1.2	5.2 ± 1.2
LVEDV (mL/m^2^)	53 ± 9	54 ± 11
LVESV (mL/m^2^)	21 ± 4	22 ± 5 *
Stroke volume (mL/m^2^)	30 ± 6	30 ± 6
LVEF (%)	60 ± 3	59 ± 3 *
S' (cm/s)	8.1 ± 1.4	8.1 ± 1.6
Biplane GLS (%)	-20.3 ± 1.7	-19.3 ± 1.8 *
Triplane GLS (%)	-20.0 ± 1.6	-19.1 ± 1.8 *
Ssr (s^-1^)	-1.4 ± 0.2	-1.4 ± 0.2
Radial strain (%)	41.5 ± 10.2	41.3 ± 10.6
Circumferential strain (%)	-21.8 ± 3.4	-20.7 ± 3.1 *

eGFR = estimated globular filtration rate; LVEDV = left ventricular end diastolic volume; LVESV = left ventricular end systolic volume; LVEF = left ventricular ejection fraction; GLS = global longitudinal strain; Ssr = systolic strain rate;

* p<0.05 compared with baseline value

Follow up echocardiograms were performed 95 ± 18 days following the baseline study, within 7 days of completion of anthracycline chemotherapy. No patient developed significant valvular / pericardial disease or heart failure early after anthracycline chemotherapy.

### Left ventricular systolic function

Clinical and echocardiographic parameters before and after anthracycline chemotherapy are listed in Table 2. The LV end systolic volume increased slightly post anthracycline therapy (p = 0.02), with no change in LV end diastolic volume (p = 0.07). LVEF and biplane GLS were measured in all patients before and after anthracycline therapy (n = 140), whilst triplane GLS could only be measured in 135 (96%) patients. Global radial and circumferential strain was obtained in 133 (95%). LVEF (p = 0.04), biplane GLS (p<0.001), triplane GLS (p<0.001) and global circumferential strain (p = 0.001) decreased significantly after anthracycline therapy. Global radial strain, systolic strain rate and S’ velocity were unaltered. Whilst there was a statistically significant reduction in LVEF after anthracycline therapy, no patients developed cardiotoxicity (i.e. reduction in LVEF >10% to <53%) [10].

Subclinical LV dysfunction, defined as >11% reduction in triplane GLS, occurred in 29 patients (22%). Subclinical LV dysfunction was not associated with any cardiovascular risk factors (family history, hypercholesterolaemia, smoking, hypertension, diabetes), clinical characteristics (age, heart rate, mean arterial blood pressure, body surface area), type of anthracycline therapy or dose. The incidence of subclinical LV dysfunction by GLS did not correlate with the percentage change in LVEF (p = 0.05). In the sub group with >11% reduction in GLS, there was no significant difference in LV end diastolic volume (p = 0.98) or LV end systolic volume (p = 0.37) compared to before treatment. The incidence of subclinical LV dysfunction was not associated with the percentage change in global circumferential (p = 0.39) or radial strain (p = 0.57).

Nine patients were receiving either an angiotensin converting enzyme inhibitor (perindopril, ramipril, and lisinopril) or a beta blocker (atenolol, propranolol) prior to chemotherapy. There were no significant differences in LVEF, GLS, radial or circumferential strain before or after anthracyclines between this group and those not receiving these medications.

Patients receiving doxorubicin had similar LVEF compared to epirubicin after therapy (59 ± 2% vs. 60 ± 2%, p = 0.23). Additionally there was no significant difference in triplane GLS (-19.1 ± 1.6% vs. -19.1 ± 2.2%, p = 0.96) after therapy between the drug types. 21% (21/102) in the doxorubicin group and 24% (8/33) in the epirubicin group had subclinical LV dysfunction by triplane GLS (p = 0.63).

**Fig 4:**
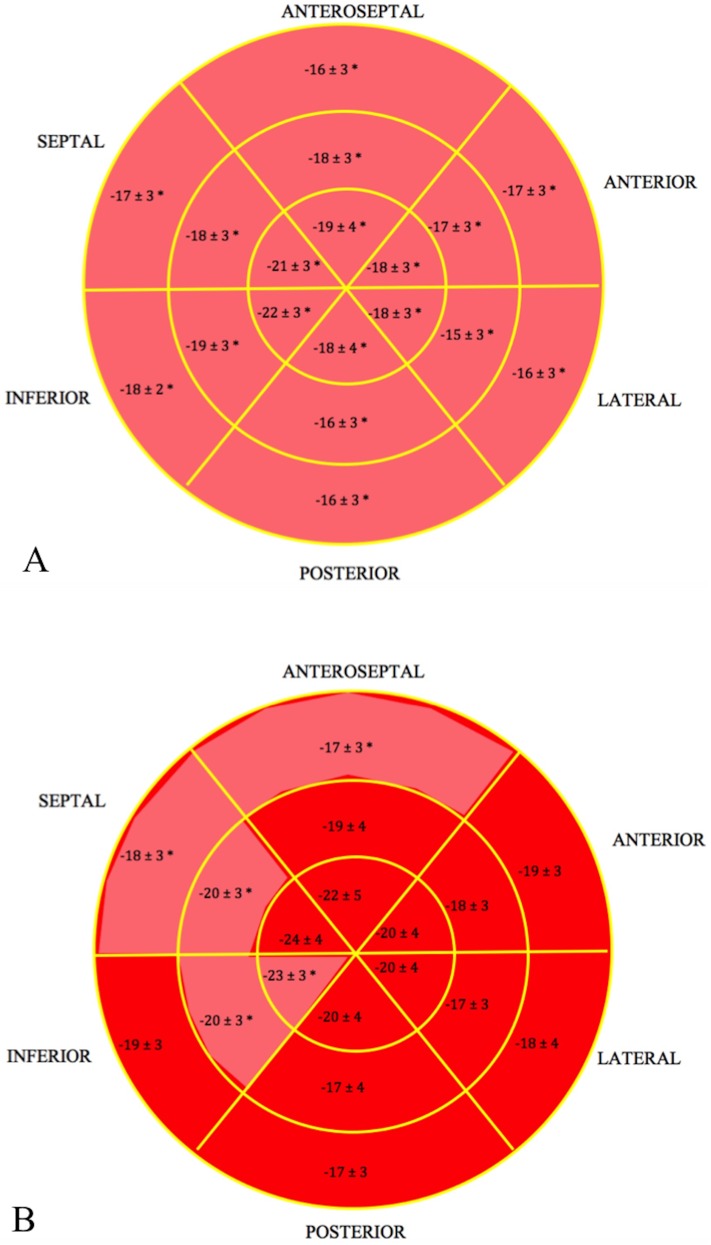
Bullseye map of GLS after anthracycline therapy. A. in the group with subclinical LV dysfunction (reduction in GLS>11%) and B. in the group without subclinical LV dysfunction. * p<0.05 compared to before anthracycline therapy.

In the group with subclinical LV dysfunction by GLS all 18 regional segments had significantly reduced strain after therapy (Fig 4A), while up to five regional segments had reduced strain after compared to before therapy in the group without reduced GLS (Fig 4B). Furthermore, in the group with subclinical LV dysfunction 9 ± 2 (58 ± 17%) regional segments had a reduction in strain by >11%, compared to 5 ± 2 (29 ± 15%) regional segments in the group without dysfunction (p<0.001).

The intra and inter-observer intraclass coefficients for LVEF were 0.77 and 0.84, for triplane GLS were 0.95 and 0.86, for systolic strain rate were 0.83 and 0.70,

### Left atrial (LA) volumes and diastolic function

LA volumes did not change post anthracycline therapy (Table 3). However, 32% of patients did have a ≥10% increase in LA maximum volume after therapy, whilst 36% had a ≥10% increase in LA minimum volume. An increase in LA maximum or minimum volume was not associated with subclinical LV dysfunction (p = 0.18 and p = 0.38).

**Table 3:** Left atrial (LA) volumes.

	Before anthracycline chemotherapy	After anthracycline chemotherapy
LA maximum volume _indexed_ (mL/m^2^)	27.1 ± 6.2	26.9 ± 5.9
LA minimum volume _indexed_ (mL/m^2^)	12.2 ± 3.6	12.3 ± 3.3
Pre 'a' wave volume _indexed_ (mL/m^2^)	18.6 ± 4.9	18.6 ± 4.8
LA total emptying volume _indexed_ (mL/m^2^)	14.7 ± 4.2	14.6 ± 3.6
LA total emptying fraction (%)	54 ± 10	54 ± 7
Conduit volume _indexed_ (mL/m^2^)	15.3 ± 6.0	15.8 ± 6.1
Passive emptying volume _indexed_ (mL/m^2^)	8.4 ± 3.1	8.3 ± 2.7
Passive emptying fraction (%)	32 ± 8	31 ± 8
Active emptying volume _indexed_ (mL/m^2^)	6.3 ± 2.1	6.3 ± 2.5
Active emptying fraction (%)	34 ± 7	34 ± 7

Diastolic parameters are listed in Table 4. Peak E velocity, E/A ratio, pulmonary vein diastolic velocity, E’ velocity and early diastolic strain rate (Esr) were significantly reduced post anthracycline therapy. Atrial fraction and late diastolic strain rate (Asr) increased post treatment. Diastolic function grade was more prevalent with ‘impaired diastolic function’ increasing from 46% to 57% (p<0.001) and normal diastolic function decreasing from 53% to 42% (p<0.001). Fourteen percent (20/140) of patients had an increase in diastolic dysfunction grade following anthracycline treatment. Patients with >11% reduction in systolic GLS had a significant increase in diastolic dysfunction grade compared to those without changes in GLS (30% vs. 11%; p = 0.04). Furthermore, there was a greater percentage change in early diastolic strain rate (p<0.001), E velocity (p<0.001), and the E' velocity (p = 0.007) in the group with subclinical LV dysfunction compared to those without. Multivariate regression analysis of these independent predictors (percentage change in early diastolic strain rate, E velocity and the E’ velocity) demonstrated that the percentage change in early diastolic strain rate (p = 0.004) and E velocity (p = 0.01) remained predictive of >11% reduction in systolic GLS.

**Table 4:** Left ventricular diastolic parameters.

		Before anthracycline chemotherapy	After anthracycline chemotherapy
**Mitral inflow**	E Velocity (m/s)	0.68 ± 0.14	0.64 ± 0.13 *
	A Velocity (m/s)	0.63 ± 0.14	0.64 ± 0.16
	E/A ratio	1.13 ± 0.32	1.04 ± 0.29 *
	Deceleration time (ms)	212 ± 40	212 ± 44
	Atrial fraction (%)	36.0 ± 7.0	39.6 ± 8.5 *
**Pulmonary vein flow**	Systolic velocity (m/s)	0.50 ± 0.14	0.49 ± 0.16
	Diastolic velocity (m/s)	0.41 ± 0.12	0.38 ± 0.12 *
	Atrial reversal velocity (cm/s)	0.27 ± 0.07	0.27 ± 0.09
**Tissue Doppler imaging**	E’ _average_ velocity (cm/s)	9.1 ± 2.4	8.4 ± 2.6 *
	E/E’ _average_ ratio	7.6 ± 2.3	7.7 ± 2.5
	A’ _average_ velocity (cm/s)	9.0 ± 2.4	9.1 ± 2.2
**LV diastolic grade**	Normal (%)	53	42 *
	Impaired (%)	46	57 *
	Pseudonormal (%)	1	1
**Strain rate**	Esr (s^-1^)	1.54 ± 0.23	1.48 ± 0.23 *
	E/Esr (s^-1^)	0.44 ± 0.10	0.44 ± 0.10
	Asr (s^-1^)	0.98 ± 0.24	1.04 ± 0.22 *

* p<0.05 compared with baseline value

Esr = early diastolic strain rate; Asr = late diastolic strain rate

## Discussion

In the present study we evaluated LV systolic function prior to, and within seven days of completing anthracycline based chemotherapy using LVEF and strain analysis, in 140 consecutive breast cancer patients. The principal findings of the study were; 1) longitudinal and circumferential strain were significantly reduced after anthracycline therapy, despite clinically insignificant changes in LVEF, 2) LV subclinical dysfunction, defined as a >11% reduction in GLS, occurred in 22% of the cohort, 3) reductions in global strain were induced equally by doxorubicin and epirubicin, 4) in patients with >11% reduction in GLS, greater segmental strain reduction was evident and 5) increased occurrence of diastolic dysfunction occurred with subclinical systolic dysfunction.

This study includes a relatively large number of breast cancer patients treated only with anthracycline chemotherapy, and confirms the occurrence of subclinical LV systolic dysfunction post therapy as reported previously [11, 16, 17]. Anthracycline induced cardiotoxicity is caused by multiple mechanisms [18] resulting in myocardial cell death and interstitial fibrosis immediately after exposure and is dependent on the cumulative dose [10, 19]. A systematic review reported that alterations in strain occurred prior to significant changes in LVEF [20], and GLS was the most consistent measure, whilst LVEF had variability with clinically significant reductions reported only at late follow up [20]. Within the current cohort, changes indicative of LV subclinical dysfunction using GLS were observed in 22% of patients, whilst there was no evidence of cardiotoxicity by LVEF. Our results provide additional evidence that changes in global LV function are associated with greater regional involvement. Anthracycline cardiotoxicity may predominantly affect the subendocardial LV layer, which is primarily composed of longitudinal fibres [18] as measured by GLS. LVEF is largely a measure of radial function; thus alterations in LVEF may not be apparent until substantial myocardial involvement occurs.

Negishi et al demonstrated that a reduction of >11% in GLS was predictive of the longer term reduction in LVEF and cardiotoxicity [14] in patients receiving trastuzumab and/or concurrent anthracyclines. In the current study, investigating only anthracycline therapy, 22% of patients had a reduction in GLS by >11% [10]. A recent study reported that LV end diastolic volume, LVEF and GLS measured post anthracycline treatment were independently associated with the development of cardiotoxicity [21]. Furthermore, higher GLS (-17% vs -12%) at the time of cardiotoxicity diagnosis was associated with subsequent improvement of LVEF [21]. Other studies have demonstrated that GLS was independently associated with all-cause mortality [22], major adverse cardiovascular events [23] and cardiotoxicity [24]. Furthermore, early reductions in LVEF and GLS after anthracycline chemotherapy persist at follow up [16, 25]. Longer term multicentre studies are required to determine the extent of early change that best predicts future cardiotoxicity, in such patients.

Doxorubicin and epirubicin are the most frequently used anthracyclines in breast cancer regimens [2]. Both agents are limited by dose-dependent cardiotoxicity, however the toxicity profile of doxorubicin may be worse than epirubicin [2]. In the current cohort there were no significant differences in LV function of both LVEF and GLS between patients receiving doxorubicin and epirubicin, although the subgroups differed in size.

The incidence of myocardial damage after anthracycline chemotherapy may be enhanced by smoking [26], preexisting cardiovascular disease, coexisting damage or individual patient genetic predisposition [10, 18]. Development of subclinical LV dysfunction in the current study was not associated with any cardiovascular risk factors or clinical parameters, specifically anthracycline drug, dose, age and body habitus, thereby highlighting the mandate for regular cardiac assessment in all patients receiving anthracyclines.

Early identification of subclinical LV dysfunction is important as this patient subgroup requires close monitoring and enables early initiation of cardioprotective agents. Treatment with an angiotensin receptor blocker (candesartan) prior to anthracycline, trastuzumab and radiotherapy was demonstrated to alleviate reduced LVEF [27], in the PRADA trial, whilst there was no advantage in metoprolol (beta blocker) [27], albeit in a small number of patients. However, others have shown early implementation of beta blockers improved GLS in patients with subclinical LV dysfunction receiving anthracyclines, trastuzumab or both [7]. Furthermore, Seicean et al reported in a large observational study that incidental and continuous incidental beta blocker treatment reduced the onset of new heart failure in patients treated with anthracyclines and trastuzumab [28]. An in-vitro mice study showed a link between the tyrosine kinase receptor and beta adrenergic systems which may explain the treatment benefits of beta blockers particularly in patients receiving trastuzumab (tyrosine kinase receptor antibody) [29]. In the current cohort, only few patients were receiving beta blocker or angiotensin converting enzyme inhibitor before anthracycline therapy; no significant differences were observed immediately after treatment. Further studies are needed to validate the cost effectiveness and benefits of beta blockers, angiotensin converting enzyme inhibitors or a combination of agents administered prior as indicated by subclinical LV dysfunction in preventing heart failure.

Diastolic dysfunction has been previously reported following anthracycline chemotherapy [26, 30–33], albeit in small groups. The development of diastolic dysfunction has been reported in up to 57% of patients treated with anthracycline chemotherapy and were independently predicted by age and body mass index [31]. Furthermore, we have also previously reported reduced early diastolic strain rate post anthracycline therapy [32].

LV diastolic grade was worse in the current cohort after anthracycline chemotherapy. Importantly, diastolic dysfunction was more prevalent in the sub group with >11% reduction in GLS, demonstrating the close association between systolic and diastolic function. In fact, the only predictors of >11% reduction in GLS was early diastolic strain rate and the mitral peak E velocity. Altered early LV relaxation resulted in augmented active atrial contraction with increased atrial fraction and late diastolic strain rate, to compensate for the decreased volume transfer during early diastole. These changes, however, were only evident by Doppler parameters and not atrial volume measurements, which are more reflective of the chronicity of LV diastolic dysfunction. The monitoring of diastolic function is of particular importance as alterations in diastolic function with relatively preserved systolic function can also result in heart failure.

### Limitations

This single-centre study (n = 140) is significantly larger than previous single-centre studies. Due to cost constraints cardiac biomarkers could not be obtained in the majority of the cohort. The study was limited by the short duration of patient follow-up, and therefore the longer term impact of early reduction in GLS is uncertain; longer term follow up is ongoing to determine the significance of these early observations. Another imaging modality may have provided additional information about LV systolic function; the use of MRI was beyond the scope of the current study. 3D echocardiography, contrast imaging and stress testing were not performed in the current cohort. Image acquisition and measurements were performed using equipment from a single vendor (GE); therefore values are not comparable with non-GE machines [34]. Measurement of GLS is limited by 2D image quality and temporal resolution. As reported, 11 patients were excluded due to poor image quality following breast reconstruction.

## Conclusion

Subclinical LV dysfunction by GLS was observed in 22% of patients. There was an increase in prevalence of LV diastolic dysfunction in the subgroup with >11% reduction in GLS. No clinical risk factors were predictive of subclinical LV dysfunction, thus emphasizing the need for all patients to be monitored for deterioration in both systolic and diastolic function following anthracycline treatment. Longer term follow up is needed and is presently ongoing to determine the clinical importance of these early findings.

## Supporting information

S1 File De identified data set. (XLSX)
